# Detecting new neurodegenerative disease genes: does phenotype accuracy limit the horizon?

**DOI:** 10.1016/j.tig.2009.09.008

**Published:** 2009-11

**Authors:** David C. Samuels, David J. Burn, Patrick F. Chinnery

**Affiliations:** 1 Center for Human Genetics Research, Department of Molecular Physiology and Biophysics, Vanderbilt University Medical Center, Nashville, TN 37232, USA; 2 Clinical Ageing Research Unit, Institute for Ageing and Health, Campus for Ageing and Vitality, Newcastle-upon-Tyne, NE4 5PL, UK; 3 Mitochondrial Research Group, Institute for Ageing and Health & Institute of Human Genetics, The Medical School, Newcastle University, Newcastle-upon-Tyne, NE2 4HH, UK

Neurodegenerative diseases present a major health challenge to the ageing population, and most are thought to arise through a complex interplay between inherited genetic variation and environmental triggers. Although rare monogenic forms of common neurological disorders exist, these account for <5% of the total number of cases. Large-scale genome-wide association studies (GWAS) are starting to have some success in identifying the major risk alleles involved in several common neurodegenerative disorders [1]. However, as research moves to the next phase of GWAS, one needs to ask whether more emphasis should be placed on phenotypic accuracy, rather than simply increasing sample sizes.

The diagnosis of a late-onset neurological disease generally relies heavily on the clinical description provided by an experienced neurologist. Specific diagnostic tests are rare, and several autopsy case series have demonstrated a diagnostic error rate approaching ∼10%, even in expert hands [2,3]. Diagnostic revision also occurs in ∼1/3 of cases [4,5]. Phenotypic misclassification reduces the power to detect a statistical association between a phenotype and specific allele for a given sample size [6–9]. Although this is primarily a clinical issue, the genetics community should be concerned; it is also relevant for any human disease where the clinical classification is not 100% accurate [8,9].

There have been several approaches to try and deal with the issue of diagnostic inaccuracy in neurodegenerative diseases (Box 1). *In silico* modelling has shown that increasing the sample size counterbalances diagnostic error [10], but that the relationship between statistical power and diagnostic accuracy is not linear; in addition, the sample size required to generate reasonable power increases dramatically with reduced diagnostic accuracy [10]. For strong genetic effects, the precise diagnosis might not be a key issue. For example, even when 15% of cases are incorrectly classified as Alzheimer disease, a study of 500 cases and 500 controls would have >70% power to detect the well-established association with the ɛ4 *APOE* allele (Online Supplementary Material Figure S1). However, the detection of hitherto unknown modest disease associations at the whole-genome level presents a greater challenge [11]. For common genetic variants exerting a modest effect [where the genome relative risk (GRR) is 1.3], a diagnostic error rate of ∼2% has little effect on statistical power (Figure 1a). However, >2% diagnostic error has a dramatic effect on power, especially when attention is drawn to lower-penetrance alleles (i.e. GRR ≤1.1), as proposed for many complex traits. This is further compounded when less frequent but equally plausible genetic variants (with a minor allele frequency ≤10%) are considered, which are highly sensitive to diagnostic errors (Figure 1b). Studies of rarer disease phenotypes (affecting <1 in 1000 adults) present an even greater challenge (Figure 1c). This includes well-recognized disorders [such as amyotrophic lateral sclerosis (ALS), or progressive supranuclear palsy (PSP)], or clinical subgroups of common disorders (such as cases of Parkinson's disease with dementia), where distinct genetic factors are thought to modulate the phenotype.

So should the sample size simply be increased, or should the samples be chosen more carefully by more accurate phenotyping? Reduced diagnostic accuracy is associated with an increase in the number of samples required for a study of equivalent power, irrespective of the GRR (Figure 1d). Thus, to achieve the same effect, investigators could either improve the phenotypic accuracy and remove false-positive cases from an existing cohort, or they could inflate the number of cases by up to 400-fold to compensate for the diagnostic errors of up to 20% (Figure 1d). We argue that it is more cost effective to improve phenotypic accuracy than it is to increase the sample size. For example, even when considering alleles with a modest effect (GRR = 1.3), increasing diagnostic accuracy from 90% to 95% would reduce the number of affected individuals needed by threefold while maintaining the same power. For alleles with weaker effects, the absolute number of cases required for 95% power at *P* < 1 × 10^−7^ will be in excess of 1 million for samples diagnosed with <99% accuracy (Online Supplementary Material Figure S2). This might never be feasible, especially when genetic and environmental variations across the globe are considered. As rarer risk alleles for less common diseases are sought, practical problems with accurate phenotyping will become a major limiting factor to these studies.

Although quantifying phenotypic accuracy can, in itself, present challenges, there are good examples where the cost effectiveness can be evaluated. Single photon emission computed tomography (SPECT) brain imaging with the ligand (123)I-2beta-carbometoxy-3beta-(4-iodophenyl)-N-(3-fluoropropyl)-nortropane((123)I-FP-CIT, has a specificity of ∼100% for a diagnosis of degenerative parkinsonism [12], although this test cannot reliably differentiate Parkinson's disease from atypical parkinsonian disorders, such as PSP. Furthermore, in the differential diagnosis of dementia, an abnormal FP–CIT scan has a specificity of 90% for excluding non-Dementia with Lewy bodies (DLB) dementia [13]. Intriguingly, each FP–CIT scan costs about the same as a state-of-the art genome-wide SNP array (∼£500). For biologically plausible risk alleles with a minor frequency of 10% conferring a GRR of 1.3, increasing diagnostic accuracy by 10% would mean genotyping ∼8000 rather than ∼750 000 cases. For relatively uncommon neurodegenerative diseases, such as ALS (which has a prevalence ∼1 in 20 000) and PSP (affecting ∼5 in 100 000), it might never be possible to assemble cohorts with >100 000 cases from a genetically homogeneous population; studies of uncommon alleles with modest effects will only be possible with an exceptionally high diagnostic accuracy, placing greater emphasis on autopsy-based series. This also applies to phenotypic subcategories of more common phenotypes if distinct genetic mechanisms are postulated.

Crucially, the same level of accuracy is not required for control subjects, where clinical misclassification of an affected individual as unaffected has less impact on statistical power [10]. This is reassuring for late-onset neurodegenerative disease, where there is always the risk that a currently asymptomatic individual will develop clinical features in the future. Providing the disease is rare (<10% of the population), the age-related penetrance is not a major concern [10].

Now that GWAS has helped to identify the ‘low hanging fruit’ in complex disease (i.e. common alleles with strong genetic effects), the emphasis shifts to the detection of the ∼20–100 low penetrance disease-specific variants thought to underpin most common complex traits, some of which might contribute to interindividual phenotypic variability. To accomplish this, the approach needs to change. Although current aspirations have been fuelled, in part, by technological advances in molecular genetics, horizons for some diseases will be restricted by inaccurate clinical diagnosis. Several other factors currently limit one's ability to detect new neurodegenerative disease genes, including the limited resolution of current studies to detect rarer genetic variants, epistatic genetic and epigenetic effects, and the role of a changing environment. Some of these issues will be difficult to resolve, and also costly, so perhaps the emphasis should now move towards improving phenotypic accuracy, because this will enrich the yield using current molecular approaches.

## Figures and Tables

**Figure 1:**
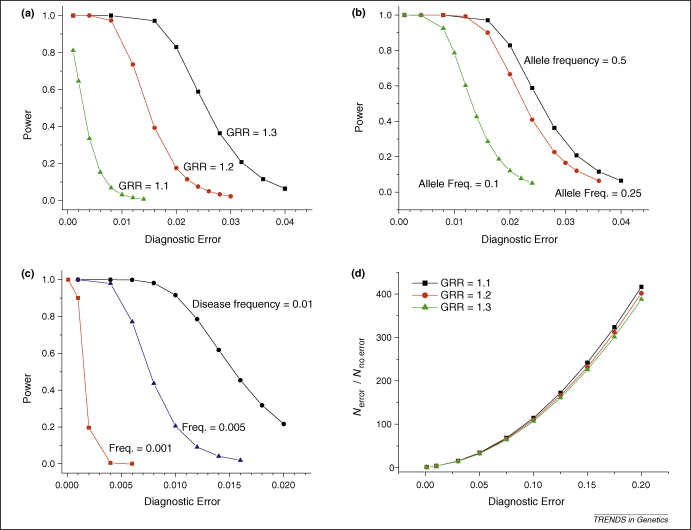
Power to detect a genetic association in the context of diagnostic errors. In each example, the probability of affected individuals being classified as controls is 1 × 10^−5^. Varying this parameter has negligible impact on power and/or optimal sample size for diseases that are present in <10% of the population [10]. **(a)** Power to detect an association between a common allele (allele frequency = 0.5; GRR = 1.1– 1.3 under a multiplicative model) and disease in 20 000 cases and 20 000 controls with varying degrees of diagnostic error at *P* < 5 × 10^−7^. Disease frequency = 0.01. **(b)** Power to detect an association between alleles of different frequency (0.5, 0.25, 0.1) and disease in 20 000 cases and 20 000 controls with varying degrees of diagnostic error at *P* < 5 × 10^−7^. GRR = 1.3, disease frequency = 0.01. **(c)** Power to detect an association between an allele (frequency = 0.125, GRR = 1.3) and diseases of different prevalence (0.01, 0.001, 0.0001) in 20 000 cases and 20 000 controls with varying degrees of diagnostic error at *P* < 5 × 10^−7^. **(d)** Ratio of the number of inaccurately phenotyped cases (*n*_error_) to the number of accurately phenotyped cases (*n*_no__error_) required to detect an association between an allele (frequency = 0.1, varying GRR from 1.1 to 1.3) and a disease (frequency = 0.01) with 95% power at varying degrees of diagnostic error at *P* < 5 × 10^−7^. All calculations used PAWE-PH Phenotype Edition [10].

## Box 1 Approaches to counteract phenotypic misclassification in neurodegenerative disease

• Using standardised and validated clinical diagnostic criteria
• Use of additional clinical data to substantiate the diagnosis (use of ‘endophenotypes’; such as cognitive, motor-system and behavioural rating scales, biochemical tests or imaging parameters)
• Studying prospective cohorts (thus avoiding retrospective, third-hand clinical evaluation, and enabling diagnostic re-evaluation as the disease progresses).
• Exclusively studying cases with a histological diagnosis (e.g. biopsy positive or autopsy case series).
• Increase the size of the study.
