# Supplementary material for: Detecting new neurodegenerative disease genes: does phenotype accuracy limit the horizon?
Source: Trends Genet. 2009 Nov;25(11):486–8. doi: 10.1016/j.tig.2009.09.008 (PMC2824109; doi:10.1016/j.tig.2009.09.008)
Supplement: Supplementary file 1 [file mmc1.doc]

**Supplementary material**

Detecting new neurodegenerative disease genes: does phenotype accuracy limit the horizon?

David C Samuels^1^, David J Burn^2,3^, Patrick F. Chinnery^3^

^1^Center for Human Genetics Research, Department of Molecular Physiology and Biophysics, Vanderbilt University Medical Center, Nashville, TN 37232, USA. [david.samuels@chgr.mc.vanderbilt.edu](mailto:david.samuels@chgr.mc.vanderbilt.edu)

^2^Clinical Ageing Research Unit, Institute for Ageing and Health, Campus for Ageing and Vitality, Newcastle upon Tyne, NE4 5PL.

[d.j.burn@ncl.ac.uk](mailto:d.j.burn@ncl.ac.uk)

^3^Mitochondrial Research Group, Institute of Ageing and Health & Institute of Human Genetics, The Medical School, Newcastle University, Newcastle upon Tyne, NE2 4HH, UK.

[p.f.chinnery@ncl.ac.uk](mailto:p.f.chinnery@ncl.ac.uk)

Corresponding author: Chinnery, P.F. (p.f.chinnery@ncl.ac.uk)

Conflicts of interest: none

**Supplementary figure S1.** Power to detect an association between the *APOE* ε4 allele and Alzheimer’s disease at P < 0.05 with varying degrees of diagnostic error in 500 cases and 500 controls. Based on an allele frequency of the ε4 allele of 0.14, conferring a genotype relative (GRR) of 5.5, and with a disease frequency of 0.02 [1, 2]**.** Calculations used PAWE-PH Phenotype edition [1].

**Supplementary figure S2.** Absolute number of inaccurately phenotyped cases cases required to detect an association between an allele (frequency = 0.1, varying GRR from 1.1 to 1.3) and a disease (frequency = 0.01) with 95% power and varying degrees of diagnostic error at P < 5 x 10^-7^. All calculations used PAWE-PH Phenotype edition [1].

**References**

1. Edwards, B.J.*, et al.* (2005) Power and sample size calculations in the presence of phenotype errors for case/control genetic association studies. *BMC genetics* 6, 18

2. Jarvik, G.*, et al.* (1996) Influence of apolipoprotein E genotype on the transmission of Alzheimer disease in a community-based sample. *Am J Hum Genet* 58, 191-200
